# A Survey on the Experience of Singaporean Trainees in Obstetrics/Gynecology and Family Medicine of Sexual Problems and Views on Training in Sexual Medicine

**DOI:** 10.1016/j.esxm.2019.12.001

**Published:** 2020-01-03

**Authors:** Zhongwei Huang, Derek Shangxian Choong, Adaikan P. Ganesan, Susan Logan

**Affiliations:** 1 Department of Obstetrics and Gynaecology, National University Health Systems, Singapore; 2 Institute of Molecular and Cell Biology, Agency of Science, Technology and Research, Singapore; 3 Family Medicine Service, KK Women’s and Children's Hospital, Singapore; 4 Department of Obstetrics and Gynaecology, Yong Loo Lin School of Medicine, National University of Singapore

**Keywords:** Sexual Problems, Training in Sexual Medicine, OBGYN, Family Medicine Residents

## Abstract

**Introduction:**

Asian patients may have more difficulty seeking help for their sexual problems because of a largely conservative culture. Residents from both obstetrics and gynecology (OBGYN) and family medicine (FM) departments are ideally placed to address sexual problems.

**Aim:**

This survey explored the experience of residents from OBGYN and FM in managing sexual problems and their views on training in sexual medicine (SM).

**Method:**

An anonymized questionnaire collecting data on trainee characteristics, exposure to male and female sexual problems, and training in SM was sent to all FM and OBGYN residents in Singapore. These residents had completed their medical registration with the Singapore Medical Council and were at various stages of specialty training in both FM and OBGYN residency programs in Singapore.

**Main Outcome Measure:**

Trainees’ exposure to male and female sexual problems and their views on training in Sexual Medicine.

**Results:**

The overall response from the survey was 63.5% (122/192)—54% (70/129) and 69% (52/75) of FM and OBGYN residents responded, respectively. 63% were female, with 22% being senior residents, and 55% attended Singaporean medical schools. About one quarter (30/122) of the respondents encountered patients with sexual problems at least monthly. Most would refer these patients directly to specialists, psychologists, and sex therapists. More than 80% of residents were not confident in managing sexual problems in either sex (89% for male problems; 83% for female problems). Among the recognized categories, only 30% felt confident to manage erectile dysfunction, 26% for vaginismus, while less than 10% felt confident to manage libido, arousal, or orgasm disorders. 95% of the residents agreed that SM should be part of both training curricula, with 70% and 25% suggesting at junior and senior residency, respectively. 93% of them were interested to obtain further knowledge and skills in SM through their core training curriculum and from seminars.

**Conclusions:**

This survey reported a significant number of residents in OBGYN and FM departments are regularly exposed to patients with sexual problems but lack the skills to manage them. OBGYN residents were more familiar with managing female sexual problems while FM residents tend to have more experience in male sexual problems. Almost universally, the residents in FM and OBGYN were very keen to acquire skills in SM, and the results support the incorporation of appropriate knowledge and skills into both national residency program curricula.

**Huang Z, Choong DS, Ganesan AP, et al. A Survey on the Experience of Singaporean Trainees in Obstetrics/Gynecology and Family Medicine of Sexual Problems and Views on Training in Sexual Medicine. J Sex Med 2019;8:107–113.**

## Introduction

Sexual problems are thought to be common in Asia.^1^, ^2^, ^3^, ^4^ However, the majority of research relating to sexual problems has been collected from Western populations, as sexual issues remain a taboo subject in Asian culture.^2^ Research investigating female sexual dysfunction in Asia reports rates of 20%–67% in cross-sectional urban populations from East and South East Asian populations (Korea, China, Hong Kong, Taiwan, Indonesia, Malaysia, Philippines, Singapore, and Thailand).^1^^,^^3^, ^4^, ^5^ From the few observational studies examining male sexual dysfunction, it seems that Asian men are still suffering in silence from sexual disorders despite the clear association between sexual problems and depression and diminished quality of life.^2^^,^^6^^,^^7^ In Singapore, it is estimated that the prevalence of sexual problems in male and female populations is even higher than the prevalence of depression and insomnia, estimated at 8.6% (in adults alone)^8^ and 15.3%,^9^ respectively.

Most of what we know about sexual problems come from the United States. Results from a cross-sectional study on family medical practices in Western populations suggest that up to 75% of the population may suffer from at least one speciﬁc sexual problem.^10^ Qualitative research from the United States reports that patients would like to discuss sexuality-related issues with their health-care providers but were often reluctant to do so because of fear of embarrassment or dismissal of their concerns.^11^^,^^12^ A study had shown that Asian men tend to accept sexual disorder as part of ageing, and instead of seeking appropriate treatment, they seek alternatives such as alcohol consumption to maintain their gender role.^13^ Another qualitative study in Hong Kong demonstrated that Asian women with sexual problems tend to seek medical help only when their male partners also have sexual problems. In addition, women often report sexual issues as “marital dissatisfaction” rather than as specific problems.^3^

Health-care providers, on the other hand, report a fear of “opening a can of worms,” time constraints, resources, inadequate knowledge and skills, worry about causing offense, personal discomfort, and a lack of awareness about sexual issues.^14^ This results in a reluctance to question about sexual problems.^15^ Sexual medicine (SM) has not been firmly established as a branch of medicine resulting in a lack of SM specialists^2^ and general deficiencies in training at undergraduate and postgraduate levels. A study reporting on medical schools in Malaysia had identified gaps in sexual health training,^16^ and it is likely that this reflects current undergraduate medical training across Asia. At the meeting of the International Consultation on Sexual Medicine in Paris in 2009, it was recognized that current sexual health education for undergraduate and practicing physicians was inadequate to meet the advancing science and technology and increasing patient demand for high-quality sexual health care.^17^ Leaving sexual problems and couple issues to marital counsellors may not meet the needs of these patients.

This study aims to quantify how often Singaporean residents in FM and OBGYN encounter sexual problems in their daily clinical practice as well as to explore their perceived competency in managing sexual problems. This will allow us to ascertain the personal learning needs and appraise the current training opportunities in SM in Singapore.

## Methods

An anonymized self-administered questionnaire comprising 18 questions was developed by the authors (Z.H. and S.L.) (see Appendix), modified from a similar study by Emerson et al.^18^ Z.H. and S.L. had adapted questions from the aforementioned study, whereby the questions were divided into various components: (i) basic demographics (from question 1 to question 7), (ii) assessing how often residents manage both male and female sexual problems as well as assessing their confidence in managing these problems (question 8 to question 11) with onwards referral (question 12 to question 14), and (iii) perceptions on sexual medicine training (question 15 to question 18). These questions were binary and aimed to obtain one finite response from the residents. This questionnaire was piloted on 3 OGYN residents (in their second and third years of training) and 3 FM residents (in their first and second years of training) with questions edited to enhance further clarity. The revised questionnaire was sent out as an anonymous online survey via a google document link to the program coordinators of both FM and OBGYN residency programs in Singapore for dissemination to their residents from December 2015 to January 2016. The survey was sent online twice. It was also distributed by hard copy at 3 national training meetings from February to March 2016. The residents were asked not to participate if they had filled up the survey previously either online or at an earlier national training meeting session. Comparison analyses of proportions were performed using chi-square tests using SPSS (SPSS for Windows, version 16.0; SPSS Inc, Chicago, IL). Statistical significance was defined as *P* < 0.05. Ethical approval was obtained from the National Health Group Institutional Review Board.

## Results

The total number of FM residents and OBGYN residents eligible to complete the survey was 129 and 75, respectively, based on December 2015 nominal trainee roll. The total response rate was 63.5% (122/192), with 54.3% (70/129) of FM residents and 69.3% (52/75) of OBGYN residents responding.

### Characteristics of Respondents

Almost two-thirds (77/122) of the respondents were female, which was representative of the actual proportion of females (129/192). 27% (14/52) of OBGYN residents were male, and 44% (31/70) of FM residents were male. Senior residents comprised of 22% (27/122) of the total number of respondents. 44% (23/52) and 5.7% (4/70) of senior residents were OBGYN and female, respectively. 88% (108/122) of the total number of respondents were of Chinese ethnicity, while the rest were of Malay and Indian ethnicity. More than half of the total respondents received their undergraduate medical education locally in Singapore (68/122), while the rest received their undergraduate medical education overseas (China, India, Europe/UK, Australia/New Zealand) (Table 1).

**Table 1:** Demographics of FM and OBGYN residents

Demographics	Total number of residents who respond, N = 122, N (%)	FM residents, N = 70, N (%)	OBGYN residents, N = 52, N (%)	*P* value
Average age (years ± SD) (age range)	28.5 ± 3.6 years (25–42 years)	28.6 ± 2.10 years (25–39 years)	30.5 ± 3.5 years (25–42 years)	0.0003∗
Gender	45 (36.9) male	31 (44.3) male	14 (20.0) male	0.005∗
	77 (63.1) female	39 (55.7) female	38 (80.0) female	
Experience of residents	95 (77.9) junior residents†	66 (94.3) junior residents†	29 (55.8) junior residents†	<0.0001∗
	27 (22.1) senior residents‡	4 (5.7) senior residents‡	23 (44.2) senior residents‡	
Proportion of residents who are				
1. Chinese	108 (88.5)	63 (90.0)	45 (86.5)	0.55
2. Indians	10 (8.2)	5 (7.1)	5 (9.6)	0.67
3. Malays	4 (3.3)	2 (2.9)	2 (3.8)	0.78
Undergraduate medical training	68 (55.7) trained locally	39 (55.7) trained locally	29 (55.8) trained locally	0.99
	54 (44.3) trained overseas	31 (44.3) trained overseas	23 (44.2) trained overseas	

FM = family medicine; OBGYN = obstetrics and gynecology; SD = standard deviation.

∗ Comparisons made between FM and OBGYN residents which reach statistical significance using chi-square test, where *P* < 0.05.

† Up to 3 years in residency.

‡ 4 years and more in residency.

### Experience and Perceived Competence in Managing Sexual Problems

All residents were asked how often they encounter male and female sexual problems during their clinical practice. About 5% (5/122) never encountered any sexual problems in their clinical practice to date. Almost 70% (85/122) of the residents encounter patients with sexual problems rarely (1–2 encounters per year) with no differences found between specialties (*P* = 0.38). Nearly a quarter (30/122) of the residents encounter patients with sexual problems often (1–2 encounters per month) with no difference between FM and OBGYN residents (13.7% [13/70] vs 32.7% [17/52], respectively, *P* = 0.07). Only 1.6% (2/122) of the residents encounter sexual problems frequently (1–2 encounters per week).

Almost 90% (109/122) described a lack of confidence in managing male sexual problems. More OBGYN residents (98.1% [51/52]) lacked confidence in this area than FM residents (82.9% [58/70], *P* < 0.01). For female sexual problems, 83.6% (102/122) of residents lacked confidence, with no difference found between the 2 groups (*P* = 0.08). There was no difference in the confidence level to handle sexual problems in graduates from local medical schools or overseas medical schools.

Regarding erectile dysfunction, 30.3% (37/122) of the respondents felt competent to manage. A significantly higher proportion of FM residents (44.3% [31/70]) felt competent in this area than OBGYN residents (11.5% [6/52]) (*P* < 0.01). Few felt competent in managing male ejaculatory problems (8.2%) but more from FM (12.9% [9/70]) than from OBGYN residents (1.9% [1/52], *P* = 0.03). In contrast, the proportion of OBGYN residents who felt competent in managing vaginismus, female sex drive, arousal problems, and orgasm problems was significantly higher than that for the FM residents, yet less than 20% were competent in areas other than vaginismus (Table 2). FM and OBGYN residents had unanimously chose to refer patients with sexual problems to gynecologists and urologists first and foremost, followed by sex therapist, psychologist, psychiatrist, and lastly marriage counsellor.

**Table 2:** Perceived competence of FM and OBGYN residents on managing specific sexual problems

Sexual problem	Total residents, N = 122, N (%)	FM residents, N = 70, N (%)	OBGYN residents, N = 52, N (%)	*P* value
Male erectile dysfunction	37 (30.3)	31 (44.3)	6 (11.5)	0.0001∗
Male ejaculatory dysfunction	10 (8.2)	9 (12.9)	1 (1.9)	0.03∗
Male sex drive	10 (8.2)	6 (8.6)	4 (7.7)	0.86
Male sexual arousal disorder	7 (5.7)	6 (8.6)	1 (1.9)	0.12
Male orgasm disorder	2 (1.6)	2 (2.9)	0 (0)	0.22
Vaginismus	32 (26.2)	12 (17.1)	20 (38.5)	0.008∗
Female sex drive	11 (9.0)	3 (4.3)	8 (15.4)	0.04∗
Female sexual arousal disorder	11 (9.0)	2 (2.9)	9 (17.1)	0.006∗
Female orgasm disorder	5 (4.1)	0 (0)	5 (9.6)	0.008∗

FM = family medicine; OBGYN = obstetrics and gynecology.

∗ Comparisons made between FM and OBGYN residents reach statistical significance using chi-square test, where *P* < 0.05.

### Views on Training in Sexual Medicine

Almost half (45.9%) of FM and OBGYN residents (56/122) were aware of the provision of clinical services in public health institutions which specialized in sexual problems. A significantly lower proportion from both specialties (21/122, 17.2%) knew about the provision of such clinical services in the private health-care sector (*P* < 0.001). More than 95% (117/122) felt that sexual medicine should be part of the training curriculum with no significant difference in opinions between FM and OBGYN (95.7% [67/70] vs 96.2% [50/52], *P* = 0.89).

Among residents, 70% (86/122) felt that SM should be incorporated in the training curriculum during the junior residency years (first 1–3 years of residency for FM, first 1–4 years of residency for OBGYN), while about a quarter (31/122) felt senior residency (after 3 years for FM and 4 years of residency training for OBGYN) would be more appropriate. A significantly higher proportion of OBGYN residents felt that sexual medicine training should be in the senior residency years compared with FM residents (34.6% [18/52] vs 18.6% [13/70] *P* = 0.045). Only a minority, 2.5% (3/122) of residents, preferred to receive SM training after the completion of residency.

Both groups of residents were keen to receive further experience and skills in SM, with more than 90% responding positively. A higher proportion of FM residents (97.1% [68/70]) demonstrated interest in receiving further training in SM than OBGYN residents (88.5% [46/52], *P* = 0.059). The residents were given a series of training options on how they felt that SM would be taught, and this included official training program in curriculum, psychosexual seminars, lunch tutorials, formal licensure, and certification; an “official training program in the training curriculum” embedded into the residency program was reported as the most popular option by 67% (35/52) of OBGYN residents and 61% (43/70) of FM residents. Psychosexual seminars were ranked the second choice for residents from both specialties, with a significantly higher proportion of FM residents choosing this option (48.6% [34/70] vs 26.9% [14/52] *P* = 0.02). Other training options—lunch tutorials and formal licensure and certification—were less popular (17.2% [21/122] vs 16.4% [20/122]). A minority also suggested having such training in medical school, sitting in during sexual medicine clinics, and formal didactic teaching as other training options.

## Discussion

There is a paucity of studies on sexual dysfunctions in Asian populations,^5^ and our survey aimed to understand whether sexual problems are commonly presented to Singaporean FM and OBGYN residents in the public health-care sector. From our study, a large majority (>95%) of FM and OBGYN residents do encounter male and female sexual problems during their residency, with almost 1 in 4 encountering patients with sexual problems on a monthly basis.

Although residents do encounter patients with sexual problems, majority of them lack the confidence to manage these patients. FM residents had significantly more experience managing male sexual problems, but this was restricted to erectile dysfunction (30%). This would be expected as OBGYN residents only see men in subfertility clinics. Rotations can be of short duration and hence they may not be familiar in managing men with sexual problems. Similarly, while almost a third of OBGYN residents felt more competent in managing vaginismus, only a minority of FM and OBGYN residents felt competent with issues of female desire, arousal, and orgasm dysfunction. More OBGYN residents felt competent in the areas of female sexual dysfunction than FM residents (*P* < 0.05), but overall perceived competency remains low. In contrast, the increased perceived competence among FM residents on handling male erectile dysfunction likely reflects equal opportunity to see both genders and its association with cardiometabolic disease. Perceived competence in managing other male sexual dysfunctions, ranging from 0 to 17.1%, was low for both FM and OBGYN residents. It is evident that both FM and OBGYN residents in Singapore are not equipped to handle male or female sexual dysfunction other than erectile dysfunction and vaginismus, yet the perceived competency by the residents even in managing common sexual dysfunction such as erectile dysfunction and vaginismus is lacking.

A study on OBGYN residents and practicing OBGYN specialists in the United States also reported that although female sexual problems were part of the curriculum, most residents feel ill equipped to address these problems as few (8.5%) received didactic activities on female sexual function and dysfunction. Over 90% of residents felt their confidence to manage such problems would increase through formal lectures, patient observations, and online modules.^19^ Similarly, a study from Brazil reported that the majority of national OBGYN residents enrolling in a sexuality course had little previous formal training in medical school or residency programs, and hence they did not feel confident in taking sexual histories or answering any sexuality-related questions in an obstetric setting.^20^ Portuguese general practitioners also expressed a need for training in the management of sexual dysfunction, and more than half considered that their professional degree to be inadequate in this area.^21^ In Singapore, the national training curricula for FM and OBGYN do not include management of male and female sexual dysfunctions as core subjects, which reflects a gap in the acquisition of SM knowledge locally, but this appears to be a global issue in SM training for health-care professionals.

Unsurprisingly, OBGYN residents may choose to refer men with sexual problems to urology colleagues, although it is known that many male sexual problems can be effectively managed in primary care.^22^ Furthermore, their limited exposure to male patients at work will result in a lack of confidence to handle sexual problems in men. Overall, most FM and OBGYN residents preferred to refer patients with sexual problems to a specialist. Yet, less than half were aware of specialized services for male and female sexual problems in the public or private health-care institutions provided by other health-care professionals such as sex therapists and psychologists. There is a dire need to disseminate and educate the residents on these specialized services so that appropriate referral pathways can be planned to ensure that these patients received SM care. From our survey, majority of FM and OBGYN residents realize the importance of SM in their daily practice and they agreed that SM training should be a part of the training curriculum. This study will provide evidence to propose to the specialist training and accreditation bodies locally that SM should be incorporated into the training curriculum of FM and OBGYN residency programs.

This is one of the first surveys that are conducted on FM and OBGYN residents in Singapore with their views on handling patients with sexual problems and ascertaining their need for SM training. A significant strength of this anonymous survey was the participation of female Asian residents, who were representative of all FM and OBGYN residents in Singapore. Furthermore, the proportion of female-to-male residents from FM and OBGYN was similar between the responders and the nonresponders. The survey also examined all types of sexual dysfunctions the residents may encounter during routine clinical practice, which offered a first glimpse on the frequency and types of sexual problems encountered in Singapore clinical practice. In addition, our approach resulted in response rates that were higher than expected for internal surveys,^23^ boosting the validity of the results obtained from this survey.

There were a number of limitations for this study, and it was not possible to exclude selection bias as not the whole cohort responded and those nonresponding OBGYN residents may be less interested in SM as it is a nonsurgical discipline. It may not be generalizable to all FM/OBGYN residents, residents in other specialties and male residents. It was also not possible to compare the responders to nonresponders because of the anonymity of the survey and the questionnaire did not allow for free text comments.

Despite the limitations discussed, the findings of our survey do provide stakeholders in postgraduate education sufficient data to implement a formalized training curriculum for SM in FM and OBGYN residency programs. The majority of the residents recommended that sexual medicine should be embedded into residency training, emphasizing the principle of “education fit for purpose”. Residents from both specialties see patients with sexual problems, and most lack the knowledge and skills to manage them confidently. Further considerations for official certification and licensure to ensure competency in the clinical practice of sexual medicine for those who wish to subspecialise^24^ should be considered. The United Kingdom Genitourinary Medicine Specialty Advisory has suggested a “core” curriculum to cover knowledge that all United Kingdom Genitourinary Medicine consultants require, with credentialing after completion of specialist training for those who wish to deliver such services.^25^ It may be possible to adopt such an approach in Singapore.

At the meeting of the International Consultation on Sexual Medicine held in 2009 in Paris, it was recommended that sexual health curricula need to be integrated from undergraduate medical education to postgraduate training in various specialities.^26^ The aforementioned strategies can be used to form the curricula for SM training in Singapore's FM and OBGYN residency programs. Planning the SM curriculum and promotion of SM as a subspecialty training needs future research to ensure clinical relevance in the local context. Trained SM specialists play pivotal roles to educate and train the next generation of SM specialists as well as to maintain high standards of proficiency and competency to provide high-quality health care to patients with sexual problems.

Dedicated trained health-care professionals in SM can play an important public health role in assisting couples with intimacy issues. This will aid to improve these couples' marital lives, reduce divorce rates, and perhaps even boost fertility rates. Anecdotally, Durex's global sex survey's findings suggest that Singapore is ranked in the 10 least sexually active countries, coinciding with decreasing fertility and birth rates in Singapore. Our survey shows that sexual problems are commonly encountered in Singapore. It is also likely that these are not exclusively seen by FM and OBGYN only, with other specialties/allied health disciplines (eg, urology, psychiatry, clinical psychology, and medical social work) likely benefiting from SM as part of their core curriculum training. It is crucial for Singapore to have a pool of competent health-care professionals to provide optimal care for patients with sexual problems. This survey will provide the ammunition stakeholders and trained SM specialists needed to discuss, revamp, and establish the SM curricula from undergraduate to postgraduate medical education in Singapore, which will create a unique localized model relevant for training and educating SM to health-care professionals in Southeast Asia and beyond.

## Statement of Authorship

**Category 1**
(a) Conception and Design
  Zhongwei Huang; Adaikan P. Ganesan; Susan Logan
(b) Acquisition of Data
  Zhongwei Huang; Derek Shangxian Choong
(c) Analysis and Interpretation of Data
  Zhongwei Huang; Derek Shangxian Choong; Susan Logan

**Category 2**
(a) Drafting the Article
  Zhongwei Huang; Derek Shangxian Choong
(b) Revising It for Intellectual Content
  Zhongwei Huang; Derek Shangxian Choong; Adaikan P. Ganesan; Susan Logan

**Category 3**
(a) Final Approval of the Completed Article
  Zhongwei Huang; Derek Shangxian Choong; Adaikan P. Ganesan; Susan Logan
