# Supplementary material for: A Survey on the Experience of Singaporean Trainees in Obstetrics/Gynecology and Family Medicine of Sexual Problems and Views on Training in Sexual Medicine
Source: Sex Med. 2020 Jan 3;8(1):107–13. doi: 10.1016/j.esxm.2019.12.001 (PMC7042166; doi:10.1016/j.esxm.2019.12.001)
Supplement: Appendix [file mmc1.doc]

**Appendix**

**Assessing GP and OBGYN residents’ views on dealing with sexual problems in the Asian context**

Target audience: ALL GP and OBGYN residents in all years of training before exit examinations in Singapore

** Required*

1. Gender *

Male

Female

2. Age * ________

3. Race *

Chinese

Malay

Indian

Other:

4. Type of trainee? *

Family Medicine

OBGYN

5. Stage of training *

Junior Residency

Senior Residency

6. Basic medical degree *

Locally - NUS

Locally - NTU

Locally - Duke-NUS

Overseas - USA

Overseas - Europe

Overseas - Australia / New Zealand

Overseas - China

Other:

7. Which institution are you based at? *

NUH (for OBGYN)

KKH (for OBGYN)

SGH (for OBGYN)

Polyclinic (for Family Medicine)

Public Hospitals (for Family Medicine)

GP clinic (for Family Medicine)

Other:

8. Have you seen any patients with sexual problems during your residency training *

Never

Rarely (average 1 – 2 per year)

Often (average 1 – 2 per month)

Always (average 1 – 2 per week)

9. Are you confident in dealing with male sexual problems? *

Yes

No

10. Are you confident in dealing with female sexual problems? *

Yes

No

11. Do you feel competent to manage the following sexual problems? *

|  | Yes | No |
| --- | --- | --- |
| Male erectile dysfunction |  |  |
| Male ejaculatory dysfunction |  |  |
| Female sex drive |  |  |
| Male sex drive |  |  |
| Female sex arousal |  |  |
| Male sex arousal |  |  |
| Female orgasm problems |  |  |
| Male orgasm problems |  |  |
| Vaginismus |  |  |

12. Where will you refer these patients if you are not competent to manage their problems? (Please list in order of preference 1 = most preferred etc.) *

|  | 1 | 2 | 3 | 4 | 5 |
| --- | --- | --- | --- | --- | --- |
| Gynaecology (female) / Urology (male) |  |  |  |  |  |
| Psychiatry |  |  |  |  |  |
| Psychology |  |  |  |  |  |
| Sex therapists |  |  |  |  |  |
| Marriage guidance |  |  |  |  |  |

13. Do you know of any specialized sexual problems clinics in Singapore’s public hospitals? *

Yes

No

14. Do you know of any specialized sexual problems clinics in Singapore’s private sector? *

Yes

No

15. Do you think sexual medicine should be part of your training curriculum? *

Yes

No

16. At what stage? *

Junior residency

Senior residency

Post residency

17. Which of the following methods will be most ideal for this purpose? (Please choose more than one) *

Open House lunch time tutorial

Psychosexual seminars

Official training curriculum

Formal certification and licensure

Other:

18. Are you interested to receive further experience and skills in sexual medicine? *

Yes

No
